# *BrDMC1*-mediated tolerance during pollen meiosis under heat stress in *Brassica rapa*

**DOI:** 10.3389/fpls.2026.1777430

**Published:** 2026-03-10

**Authors:** Xulin Wang, Jialin Guo, Gongyao Shi, Weiwei Chen, Gangqiang Cao, Baoming Tian, Luyue Zhang, Fang Wei, Zhengyu Wang

**Affiliations:** 1 Henan International Joint Laboratory of Crop Gene Resources and Improvements, School of Agriculture and Biomanufacturing, Zhengzhou University, Zhengzhou, China; 2 Tongbai Agricultural Technology Extension Center, Tongbai Bureau of Agriculture and Rural Affairs, Nanyang, China

**Keywords:** *Brassica rapa*, DMC1, DNA damage repair, high temperature, meiosis

## Abstract

High temperatures may have a substantial impact on cellular meiosis, and subsequently affects plant reproduction, development, and yield over time. In this study, using overexpressed transgenic lines, we show that *BrDMC1*, a gene involved in meiotic recombination, regulates heat tolerance during the early pollen development stage in *Brassica rapa*. According to the expression pattern analysis, *BrDMC1.A03* was not discovered at the transcriptional level, whereas *BrDMC1.A01* was highly expressed in young flower buds in *B.rapa*. The *Cis*-acting element prediction revealed that *BrDMC1.A01* contains a low-temperature responsive element, and GUS histochemical analysis revealed an increased staining ability following temperature stress. Under normal conditions, there were no significant cytogenetic or molecular differences between wild-type (WT) and overexpressed-*BrDMC1* (OE-*BrDMC1*).After 24 h of treatment at 38°C, compared with WT, OE-*BrDMC1* demonstrated dramatically increased pollen fertility, reduced aberrant chromosomal behaviors during meiosis, lowered reactive oxygen species (ROS) concentration, and boosted antioxidant enzymes SOD, POD, and CAT. Furthermore, genes involved in repair of DNA double-strand breaks (DSBs), as well as those that govern meiotic cell cycle transition, were considerably increased in OE-*BrDMC1* under high temperature stress. These findings suggest that *BrDMC1* could probably mediate heat tolerance during pollen meiosis, revealing the genetic basis for meiotic adaptation to high temperatures in *B.rapa*.

## 1 Introduction

Meiosis is an important phase in the sexual life cycle, influencing plant genetic diversity and ploidy stability. The highly conserved topoisomerase SPO11 and numerous other similar proteins facilitate the controlled production of DNA double-strand breaks (DSBs), which initiate meiotic recombination (Keeney et al., 1997; Grelon et al., 2001). SPO11 is endonucleolytically cleaved and removed by the MRX-N complex [MRE11-RAD50-XRS2 (Nbs1)] and Com1 (Sae2), followed by additional resection of the 5’ end by Exo1 to generate 3’ single-stranded DNA (ssDNA) (Borde, 2007). RPA binds to 3’ ssDNA ends to prevent degradation and increase recombinase loading (Ranjha et al., 2018). Subsequently, recombinases RAD51 or DMC1 replace RPA to bind to 3’ ssDNA ends, facilitating homologous search and single-strand invasion, ultimately forming crossover (CO) and non-crossover (NCO) products, with the former accompanied by genetic information exchange on chromosomes to generate genetic diversity and the latter resulting in limited genetic exchange (Bugreev et al., 2011; Kurzbauer et al., 2012; Yang et al., 2021). In plants, mutations in proteins involved in DSB generation and repair frequently lead to substantial meiotic abnormalities, defined by poor synaptonemal complex (SC) assembly, defective meiotic recombination (MR), and eventually reduced or lost plant fertility (Mercier et al., 2015).

High-temperature stress impacts several stages of plant reproduction, including meiosis, pollen formation, tapetal cells, reactive oxygen species (ROS) concentration, and fertilization (Endo et al., 2009; Parish et al., 2012; Djanaguiraman et al., 2018; Chen et al., 2021). Plant meiosis is quite sensitive to high temperatures. High temperatures (28°C) in the appropriate growth temperature range increase MR in *Arabidopsis thaliana* via increasing CO formation frequency (Modliszewski et al., 2018). Higher temperatures (32-34°C) could impact homologous synapsis in *A. thaliana*, reducing CO formation (De Jaeger-Braet et al., 2021). When temperatures reach the growth threshold (36-38°C), homologous chromosomal synapsis frequently fails, resulting in a dramatic decrease in MR (De Storme and Geelen, 2020). High temperature also disrupts chromosomal segregation and cytokinesis, resulting in abnormalities such as chromosome stickiness, lagging chromosomes, and micronucleus (Mai et al., 2019; Lei et al., 2020). Studies have demonstrated that ATM facilitates DSB repair under high temperature by acting downstream of the MRE11-RAD50-NBS1 (MRN) complex and functioning in a RAD51-independent but chromosomal axis-dependent way (Zhao et al., 2023). Furthermore, the tapetum plays an important role in the correct development of microspores and pollen grain maturation (Li et al., 2006, Li et al., 2011). In reaction to high temperatures during the microspore formation stage, tapetum-related genes in immature anthers are downregulated (Endo et al., 2009). Meanwhile, ROS are needed for normal cellular metabolic functions, but they are created and stored as harmful consequences of aerobic metabolism after plant exposure to abiotic stress. This causes significant intercellular biochemical damage (De Storme and Geelen, 2014; Mittler, 2017). Antioxidant enzymes and osmoprotectants can protect plants from oxidative stress (Choudhury et al., 2017; Khan et al., 2017; Wang et al., 2023).

The RecA-like recombinase family plays a key role in eukaryotic meiosis by facilitating homologous DNA search and strand exchange. Previous evolutionary analysis classified the RecA-like recombinase family into three subfamilies: Class I, the major recombinase subfamily RADα; Class II, the RADβ subfamily; and Class III, the RecA subfamily (Lin et al., 2006).DMC1 (disrupted meiotic control 1) is a member of the RADα subfamily that plays a role in meiotic recombination, DNA repair, and genomic stability, and repairs DSBs utilizing homologous chromosomes as templates during meiosis (Klimyuk and Jones, 1997; Crismani et al., 2013). Mutation or deletion of *DMC1* causes failure in SC formation and incorrect segregation of homologous chromosomes, resulting in germ cell death (Pittman et al., 1998; Chen et al., 2016). Heat stress (36-38°C) inhibits MR by lowering SPO11-dependent DSBs and changing chromosomal axis shape, and DMC1 protein abundance decreases dramatically under high temperature (Ning et al., 2021). Studies have also found that *DMC1* maintains seed germination under salt stress by regulating antioxidant enzyme activity and DNA repair gene expression, and *BrDMC1*-RNAi plants exhibit reduced antioxidant enzyme activity and germination rate (Wang et al., 2023). Previously, it was proposed in hexaploid wheat that the meiotic gene *TaDMC1-D1* is most likely a candidate gene for maintaining synapsis and crossovers under low or high temperature conditions, and subsequent experiments confirmed this hypothesis, *TaDMC1-D1* is responsible for maintaining normal crossover formation under low temperature and moderate high temperature (Draeger et al., 2020, Draeger et al., 2023). At 40°C, LIM15 (DMC1 homolog) in *lilium* is rapidly activated in anthers to stabilize the meiotic process and enhance crossover events, reflecting the functional conservation of DMC1 in responding to high temperature across different species (Ahn et al., 2025).

Meiotic recombination is a dynamic mechanism that is more sensitive to temperature fluctuations. However, how *DMC1* influences the meiotic process under high-temperature stress is unknown. In this study, we show that DMC1 can respond to extreme temperature stress. Although both WT and OE-*BrDMC1* lose pollen viability after high-temperature treatment, OE-*BrDMC1* has much higher pollen viability than WT. Consistent with the pollen fertility phenotype, OE-*BrDMC1* exhibit more normal chromosomal activities during meiosis than WT. Meanwhile, the tapetal cell and microspore growth processes in both WT and OE-*BrDMC1* are significantly affected, with no significant difference. In comparison to WT, OE-*BrDMC1* have considerably lower ROS levels and much higher antioxidant enzyme activity. Furthermore, high-temperature stress dramatically increases the expression levels of the *SPO11-1*, *ATR*, *ATM*, *RECA3*, *CYCA1*, and *OSD1* in OE-*BrDMC1*. These findings suggest that *DMC1* can positively influence heat tolerance during meiosis.

## 2 Materials and methods

### 2.1 Plant materials and growth conditions

*Brassica rapa* DH line CXL-45 and OE-*BrDMC1* plants were cultured in an artificial climate chamber under conditions of 16 h light/22°C, 8 h dark/16°C, and relative humidity of around 60%. The *Nicotiana benthamiana* was cultured under 16 h light/25°C, 8 h dark/25°C, and relative humidity of around 60%.

### 2.2 High-temperature treatment

WT and OE-*BrDMC1* blooming plants were placed in a growth cabinet at 38°C for 24 h, with 16 h of light and 8 h of darkness. All materials were treated between 10:00 and 11:00 a.m., and flower buds were collected for later studies.

### 2.3 *Cis*-acting element analysis of *DMC1* promoter

The *DMC1* promoter region was extracted using TBtools software and predicted using the Plant CARE website (http://bioinformatics.psb.ugent.be/webtools/plantcare/html/), with the results merged and analyzed. To create the pro*BrDMC1*::GUS vector, a 1730 bp sequence upstream of *BrDMC1* was cloned into the pEASY-T1 vector for amplification, then subcloned into the pCAMBIA3301 vector with XbaI and NcoI restriction sites, then transfected into *Agrobacterium tumefaciens* (EHA105) to complete vector synthesis. The created vector was infiltrated into tobacco leaves that had been grown for around four weeks. Exogenous hormones and stress treatments were applied after 48 h, and the results were seen 24 h later.

### 2.4 Cytology

Under normal conditions and high-temperature treatment, anthers from WT and OE-*BrDMC1* were fixed in Carnoy’s solution (ethanol: glacial acetic acid, 3:1). Pollen was stained with Alexander’s stain for observation (Alexander, 1969). The procedure for observing the meiotic process by the squashing method was as previously described (Zhao et al., 2023). Microspore development was observed using 4’,6-diamidino-2-phenylindole (DAPI) staining (Caselli et al., 2019). Observation of tapetal development was carried out as previously described (Qian et al., 2021). Finally, the slides were examined and photographed with an upright fluorescence microscope.

### 2.5 Determination of ROS content

O_2_^•−^ combines with hydroxylamine to create NO_2_^-^, resulting in a pink azo color containing sulfanilic acid and α-naphthylamine. The highest light absorption of this dye is at 540 nm. The A540 value can be used to quantify the sample’s O_2_^•−^ concentration. H_2_O_2_ combines with a specific chromogenic agent to produce a colored material with a distinct absorption peak at 510 nm, revealing the sample H_2_O_2_ content. OH^•^ oxidizes 2-deoxyribose to a malondialdehyde analog, which then condenses with thiobarbituric acid (TBA) to produce a colorful product with a maximum absorption peak at 532 nm, revealing the OH^•^ concentration.

### 2.6 Determination of antioxidant enzyme activities

Anthers were thoroughly pulverized with a pre-cooled mortar and pestle in 10 mL of 50 mM sodium phosphate buffer (pH 7.8) containing 1% (w/v) polyvinylpyrrolidone. The homogenate was centrifuged at 15,000 g for 10 min at 4 °C. The supernatant from the crude enzyme extract was maintained in an ice bath (Ahmad et al., 2018). The photochemical approach was used to assess the activity of superoxide dismutase (SOD; EC 1.15.1.1). The activity of peroxidase (POD; EC 1.11.1.7) was determined using a substrate combination containing 50 mM sodium phosphate buffer (pH 6.0), 5 mM guaiacol, and 0.25 mM H_2_O_2_. POD activity was evaluated after adding 0.1 mL of enzyme extract by measuring the change in absorbance at 470 nm every 40 s. Catalase (CAT; EC 1.11.1.6) activity was assessed by measuring the decrease in absorbance at 240 nm every 30 s following H_2_O_2_ breakdown.

### 2.7 Quantitative reverse transcription PCR analysis

Total RNA was isolated from unopened flower buds of WT and OE-*BrDMC1* plants grown under normal temperature conditions (22 °C) and after 38 °C treatment for 24 h, respectively. The Plant Total RNA Isolation Kit Plus (Foregene, Chengdu, China) was used to extract *B.rapa* total RNA, and the cDNA Synthesis Kit (TAKARA, Dalian, China) was used to create cDNA. We predicted the gene structure (using TBtools software) and functional domains (NCBI CDD database) of the meiotic gene copies provided for a focused analysis of the integrity of the core functional domains. Particular primers were made to solely amplify the target copies of the chosen copies. The LightCycler 480 System and qPCR Master Mix (Trans, Beijing, China) were used for qRT-PCR analysis. The following was the PCR program: 40 cycles of 95°C for 15 s, 60°C for 50 s, and a melting curve study from 72°C to 96°C were performed after 60 s at 95°C. As an internal control, the *B.rapa* β-ACTIN gene was employed. The 2^−ΔΔCt^ technique was used to calculate relative expression levels (Michaelidou et al., 2013). Using separate cDNA preparations, three biological replicates were carried out. The used primers are listed in **Supplementary Table 1**.

### 2.8 Statistical analyses

The experiment included at least three different biological and technological replicates. Data visualization and statistical analysis were carried out using GraphPad Prism 5. The significance of differences between the experimental and control groups was determined using the t-test and one-way ANOVA. Significant differences from the control are shown by **p < 0.01, and *p < 0.05.

## 3 Results

### 3.1 *DMC1* promoter region shows response to different stress conditions

**Figure 1:**
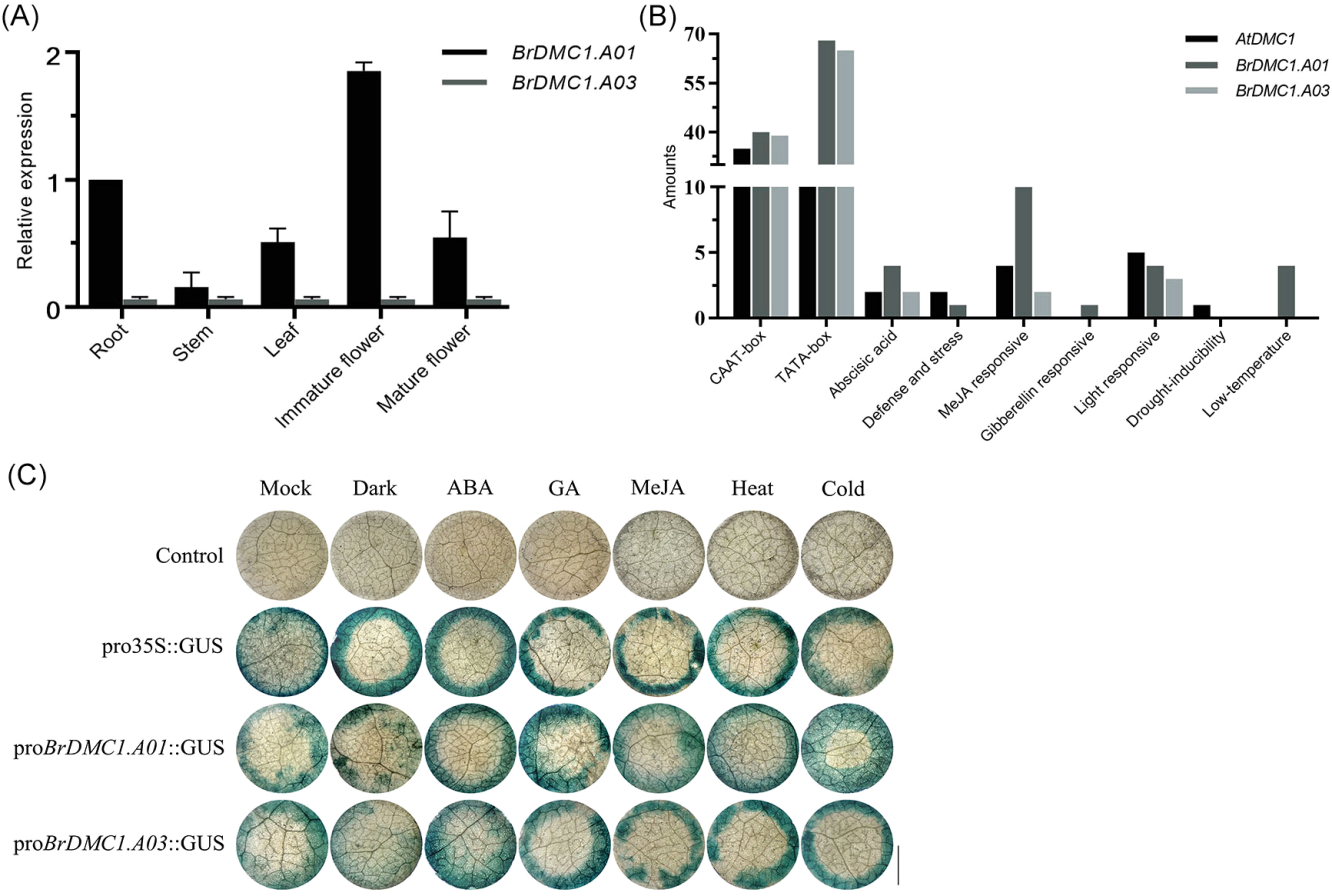
Analysis of the *BrDMC1* promoter. **(A)** RT-qPCR analysis of the expression of *BrDMC1* in various tissues of the WT. Three biological replicates were used for each sample. **(B)** Prediction of *cis*-acting elements of *BrDMC1*. **(C)** GUS staining of *N. benthamiana* leaves after different treatments to verify the activity of *BrDMC1* promoter. Bar = 0.25 cm.

Bioinformatics analysis revealed that after the triplication event, *BrDMC1* retained two copies in *B.rapa*, *BrDMC1.A01* (ID: Bra023796) and *BrDMC1.A03* (ID: Bra001890). Moreover, the genetic structure, conserved motifs, and conserved functional domains of *BrDMC1.A01* were similar to those of *AtDMC1*, whereas *BrDMC1.A03* retained only a partial structure (**Supplementary Figure 1**). The expression levels of different copies of *BrDMC1* in different tissues of plants were analyzed. The results showed that *BrDMC1.A01* was expressed in different plant tissues, and the expression level was the highest in young buds, followed by roots, leaves, and stems (**Figure 1A**). The expression level of *BrDMC1.A03* is relatively low (**Figure 1A**), and it is speculated that its biological function is lost after replication events.

*Cis*-acting element analysis of *DMC1* genes from *A.thaliana* and *B.rapa* revealed that both have light-responsive elements, defense and stress-responsive elements, GA-responsive elements, MeJA-responsive elements, and basic structural elements, CAAT-box and TATA-box, indicating promoter features (**Figure 1B**). Transient transfection of tobacco leaf cells was utilized to confirm the expression of the *BrDMC1* promoter in order to see if the promoter fragment is active and how it reacts to various hormones and stress. The results showed that tobacco leaves injected with *BrDMC1* promoter bacterial solution showed a blue color after GUS staining, indicating that *BrDMC1* is an active promoter fragment (**Figure 1C**). To see how the *BrDMC1* promoter reacted, several hormone and stress treatments were used. The result demonstrated that the blue color of leaves stained with GUS for the *BrDMC1* promoter was deepened to varying degrees following the application of ABA, GA, MeJA hormones, and high-temperature and low-temperature treatments (**Figure 1C**). This is consistent with the prediction results of promoter *cis*-acting elements, all of which are induced by distinct hormones and stress conditions.

### 3.2 Pollen fertility of OE-*BrDMC1* significantly increases under high-temperature stress

**Figure 2:**
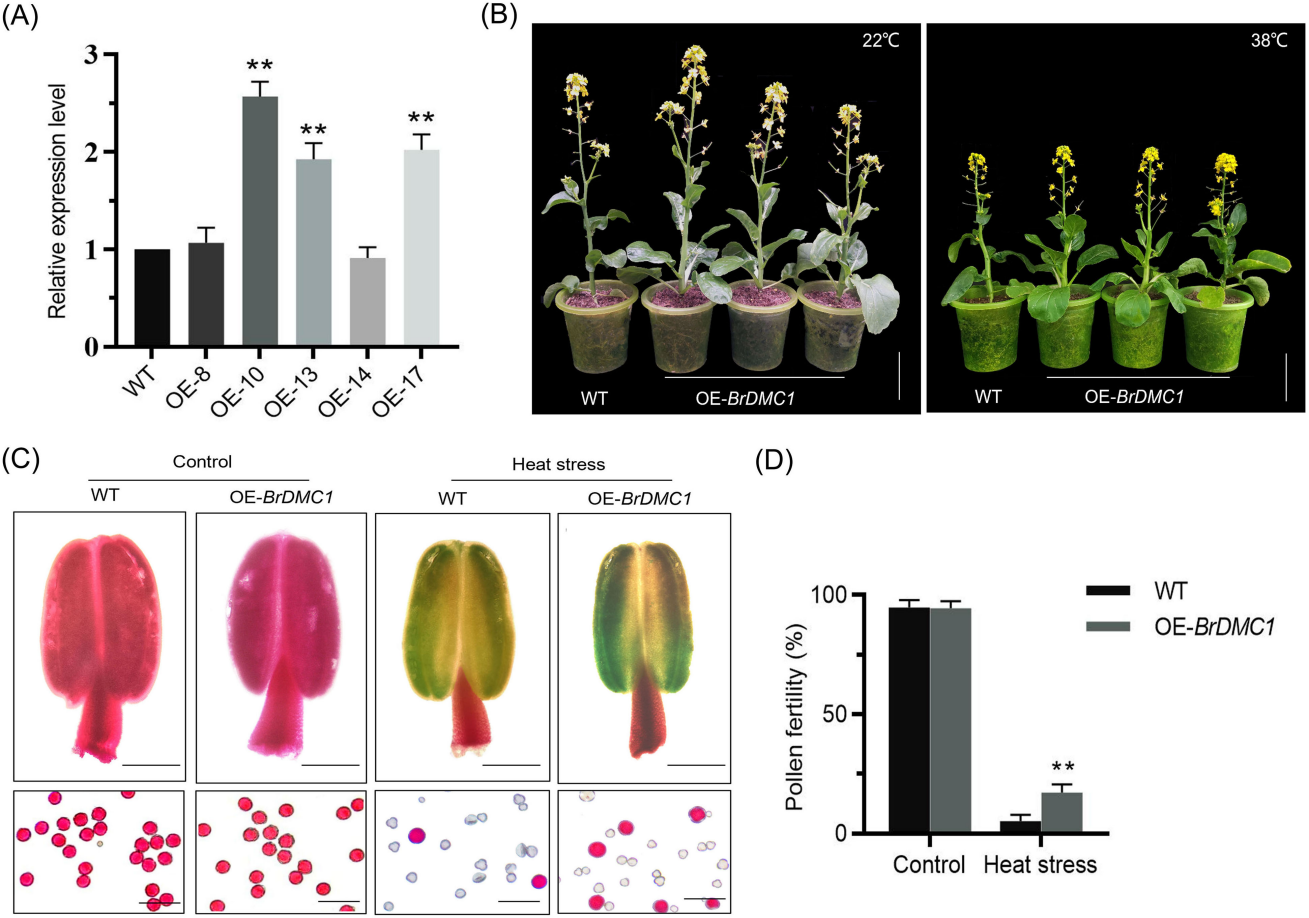
Analysis of pollen fertility of *BrDMC1*. **(A)** Detection of *DMC1* expression in WT and OE-*BrDMC1* lines under normal temperature conditions. Three biological replicates were used for each sample. **(B)** The phenotypes of WT and OE-*BrDMC1* lines (OE-10/OE-13/OE-17) under normal temperature and high-temperature conditions. Bar = 7 cm. **(C)** Anther and pollen staining of WT and OE-*BrDMC1* lines (OE-10/OE-13/OE-17) under normal temperature and heat-temperature conditions. Bar = 100 mm. **(D)** Pollen staining rates of WT and OE-*BrDMC1* lines (OE-10/OE-13/OE-17) under normal temperature and heat-temperature conditions. **p < 0.01.

In order to investigate the role of *DMC1* in plants, we created transgenic lines (OE-*BrDMC1*, OE-10/OE-13/OE-17) that expressed *DMC1* much more than the WT (**Figures 2A**, and **Supplementary Figure 2**). Plant phenotypic observation revealed that WT and OE-*BrDMC1* lines (OE-10/OE-13/OE-17) did not significantly differ in growth and development under normal conditions. Both plants displayed wilting following high-temperature stress (**Figure 2B**). Fertility studies demonstrated that under normal culture conditions, pollen grains of WT could be stained with Alexander’s stain, resulting in homogeneous pollen grain shape, and the same phenomena was found in OE-*BrDMC1* lines (OE-10/OE-13/OE-17). The majority of pollen grains in both WT and OE-*BrDMC1* lines (OE-10/OE-13/OE-17) showed abnormal discolored when exposed to high temperatures (**Figure 2C**). Further statistical examination of the stainability rate revealed that, under normal conditions, WT and OE-*BrDMC1* lines (OE-10/OE-13/OE-17) had pollen stainability rates of 97.1% (n=1085) and 97.8% (n=1054), respectively. Under high-temperature stress, the pollen stainability rate of WT was 5.3% (n=1152), whereas that of OE-*BrDMC1* lines (OE-10/OE-13/OE-17) was 17.3% (n=1023), which was significantly higher than that of WT (**Figure 2D**).

### 3.3 Abnormal meiotic chromosomal behaviors decrease in OE-*BrDMC1* under high-temperature stress

**Figure 3:**
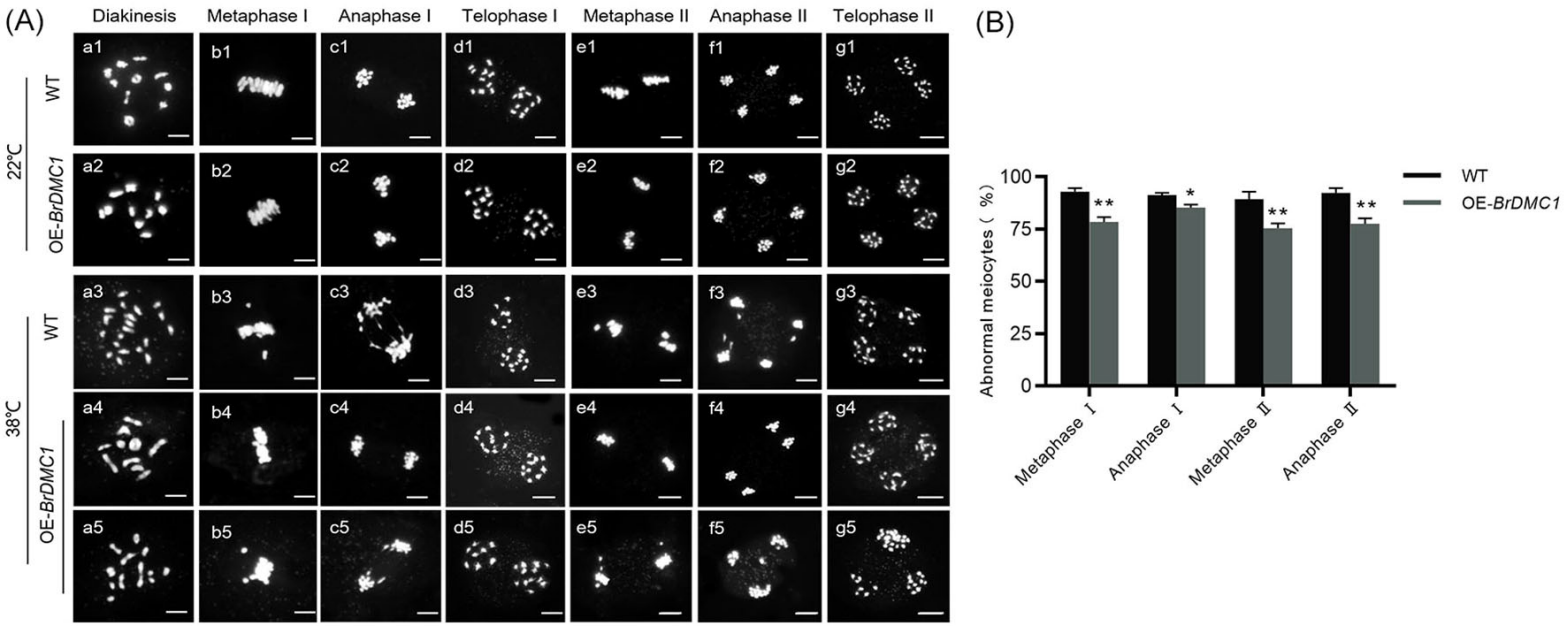
Observation of chromosome behavior during meiosis in *BrDMC1* plants. **(A)** PI-stained chromosome spreads of male meiocytes during meiosis in WT and OE-*BrDMC1* lines (OE-10/OE-13/OE-17) under normal temperature and heat-temperature conditions. Bar = 10 μm. **(B)** Percentages of abnormal meiocytes in WT and OE-*BrDMC1* lines (OE-10/OE-13/OE-17) under heat-temperature conditions. **p < 0.01 and *p < 0.05.

To explore the effect of high temperature on *B.rapa* meiosis, we observed the complete process. The findings revealed that under normal culture conditions, the complete meiotic process in WT and OE-*BrDMC1* lines (OE-10/OE-13/OE-17) was normal. Despite the fact that both WT and OE-*BrDMC1* lines (OE-10/OE-13/OE-17) displayed aberrations in the meiotic process after high-temperature treatment, OE-*BrDMC1* lines (OE-10/OE-13/OE-17) continued to exhibit normal processes. At diakinesis, WT had mostly univalents, whereas OE-*BrDMC1* lines (OE-10/OE-13/OE-17) had both univalents and bivalents (**Figure 3A**). Statistical investigation of abnormal meiotic processes revealed that at metaphase I, chromosomes were organized orderly on the equatorial plate in OE-*BrDMC1* lines (OE-10/OE-13/OE-17), whereas lagging chromosomes and chromosomal bridges were identified in 78.5% (n=123) of pollen mother cells. During anaphase I, homologous chromosomes separated, and 85.3% (n=129) of pollen mother cells formed lagging chromosomes and chromosomal bridges. At metaphase II, 74.2% (n=117) of pollen mother cells contained lagging chromosomes. In anaphase II, 77.6% (n=126) of pollen mother cells had uneven chromosomal segregation and lagging chromosomes. Finally, uneven segregation at telophase caused the creation of three or four daughter cells (**Figure 3B**). Statistical analysis of WT showed that at different stages of meiosis, the proportion of pollen mother cells with abnormalities ranged from 88.1% to 94.8% (n=118~138) (**Figure 3B**), which was significantly higher than that of OE-*BrDMC1* lines (OE-10/OE-13/OE-17), indicating that overexpression of *BrDMC1* can enhance heat tolerance during meiosis.

### 3.4 *BrDMC1* does not affect tapetal development under high-temperature stress

**Figure 4:**
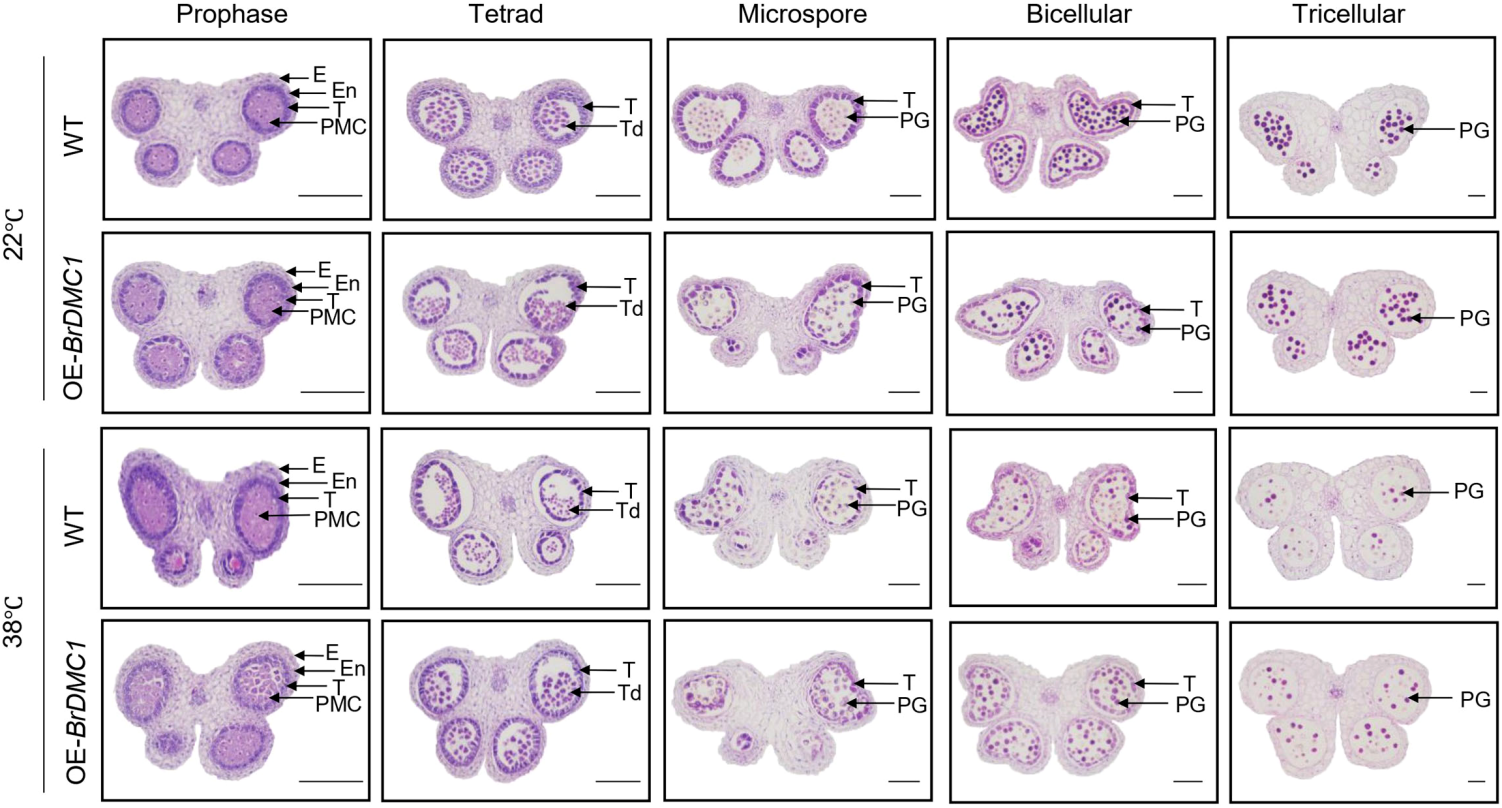
Observation of the development process of tapetum in WT and OE-*BrDMC1* lines (OE-10/OE-13/OE-17) under normal temperature and heat-temperature conditions. (E) Epidermis layer, (En) Inner layer, (T) tapetum, (PMC) Pollen mother cell, (Td) Tetrad, (PG) Pollen grain. Bar = 100 μm.

The tapetum is essential for controlling the growth of pollen. Pollen abortion results from abnormal tapetal growth, which also prevents normal pollen grain development. We observed anthers in paraffin sections to see the morphology of the tapetum during anther development. The findings demonstrated that the epidermis, endothecium, middle layer, and tapetum of WT and OE-*BrDMC1* lines (OE-10/OE-13/OE-17) did not significantly vary under normal conditions (**Figure 4**). After high-temperature stress treatment, the tapetal development process of OE-*BrDMC1* lines (OE-10/OE-13/OE-17) and WT was nearly identical to that under normal conditions during the meiotic and tetrad stages. However, at the uninucleate microspore and binucleate stages, the tapetum color diminished (**Figure 4**), indicating premature degradation, and the tapetum was totally degraded by the trinucleate stage.

### 3.5 *BrDMC1* does not affect microspore development under high-temperature stress

**Figure 5:**
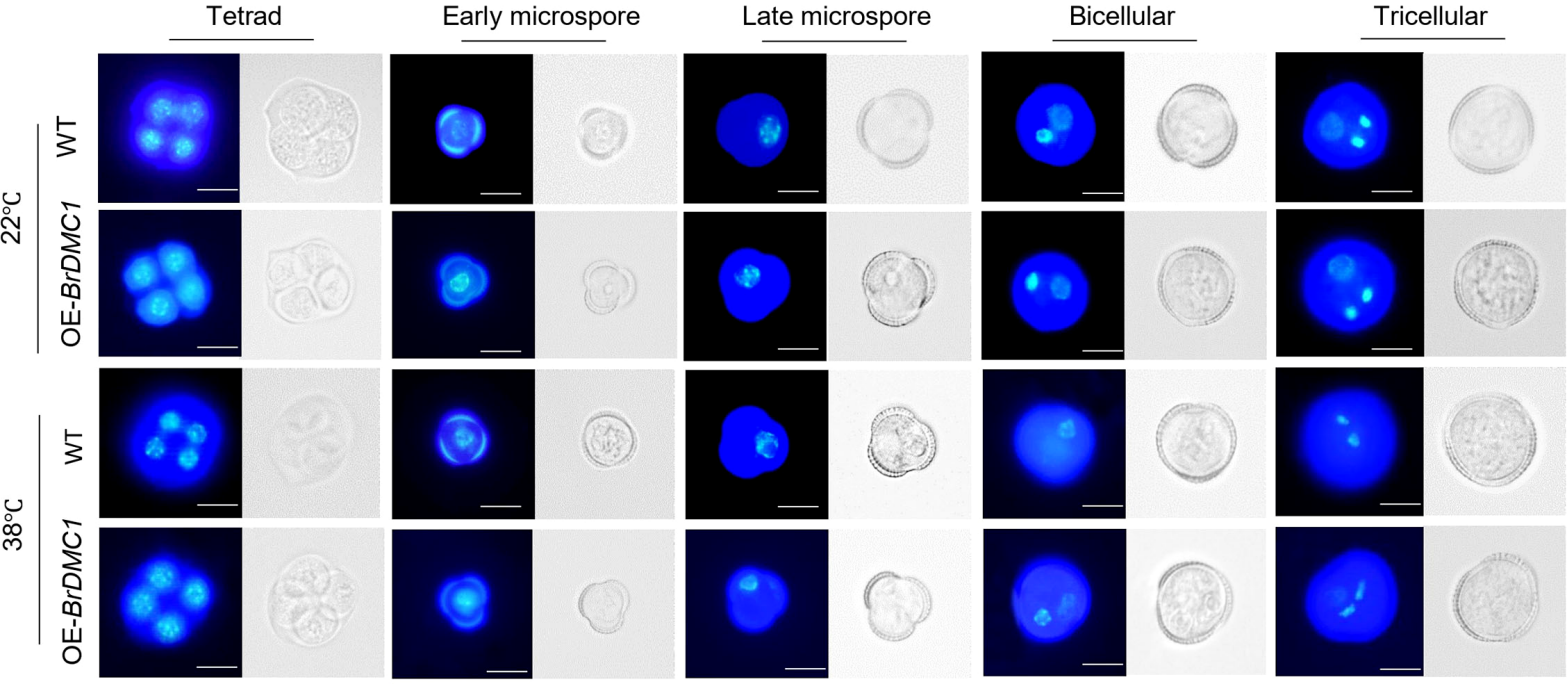
Observation of pollen development process in WT and OE-*BrDMC1* lines (OE-10/OE-13/OE-17) under normal temperature and heat-temperature conditions. Bar = 10 μm.

DAPI was used to stain microspores at various stages of development in order to better observe the microspore formation process of WT and OE-*BrDMC1* lines (OE-10/OE-13/OE-17). The findings demonstrated that both WT and OE-*BrDMC1* lines (OE-10/OE-13/OE-17) pollen mother cells underwent a typical microspore development process to produce trinucleate pollen grains under normal conditions (**Figure 5**). After high-temperature treatment, there were essentially no differences in microspore formation between WT and OE-*BrDMC1* lines (OE-10/OE-13/OE-17) at the tetrad, early uninucleate, late uninucleate, binucleate, and trinucleate stages (**Figure 5**). Furthermore, we discovered a fascinating phenomenon: after high-temperature treatment, at the binucleate stage, the vegetative nucleus was distributed diffusely throughout the cell, whereas at the trinucleate stage, the vegetative nucleus vanished, implying that high temperature primarily affects the development of the vegetative nucleus rather than the generative nucleus of plants.

### 3.6 *BrDMC1* responds to high-temperature stress via anti-oxidative damage pathway

**Figure 6:**
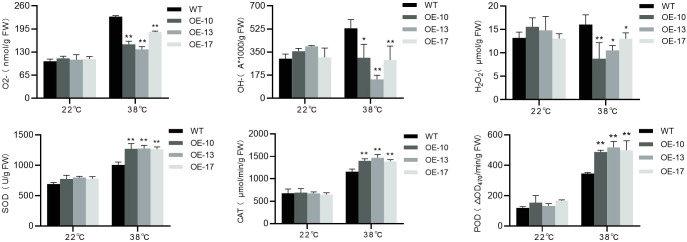
Determination of O2^•−^, H_2_O_2_, and OH^•^ content and SOD, POD, and CAT activity in WT and OE-*BrDMC1* lines (OE-10/OE-13/OE-17) under normal temperature and heat-temperature conditions. **p < 0.01 and *p < 0.05.

ROS, such as O_2_^•−^, H_2_O_2_, and OH^•^, build in plants during non-biological stress, causing oxidative damage to cells and interfering with genomic stability. Plants require antioxidant enzymes, osmoprotectants, and antioxidants to reduce the concentration of ROS and protect themselves from stress. Under normal conditions, the assessment of O_2_^•−^, H_2_O_2_, and OH^•^ contents reveals no substantial variation in the concentration of ROS between WT and OE-*BrDMC1* lines (OE-10/OE-13/OE-17) (**Figure 6**). Following high-temperature stress, the contents of O_2_^•−^, H_2_O_2_, and OH^•^ in OE-*BrDMC1* lines (OE-10/OE-13/OE-17) are much lower than those in WT, with extremely significant differences in O_2_^•−^ content and considerable differences in H_2_O_2_ and OH^•^ contents (**Figure 6**). After high-temperature stress, SOD, POD, and CAT activities in OE-*BrDMC1* lines (OE-10/OE-13/OE-17) were significantly higher than in WT (**Figure 6**). The results show that OE-*BrDMC1* lines (OE-10/OE-13/OE-17) exhibit lower oxidative damage and are more resistant to high-temperature stress.

### 3.7 *BrDMC1* responds to high-temperature stress through DNA repair pathway and cell cycle transition

**Figure 7:**
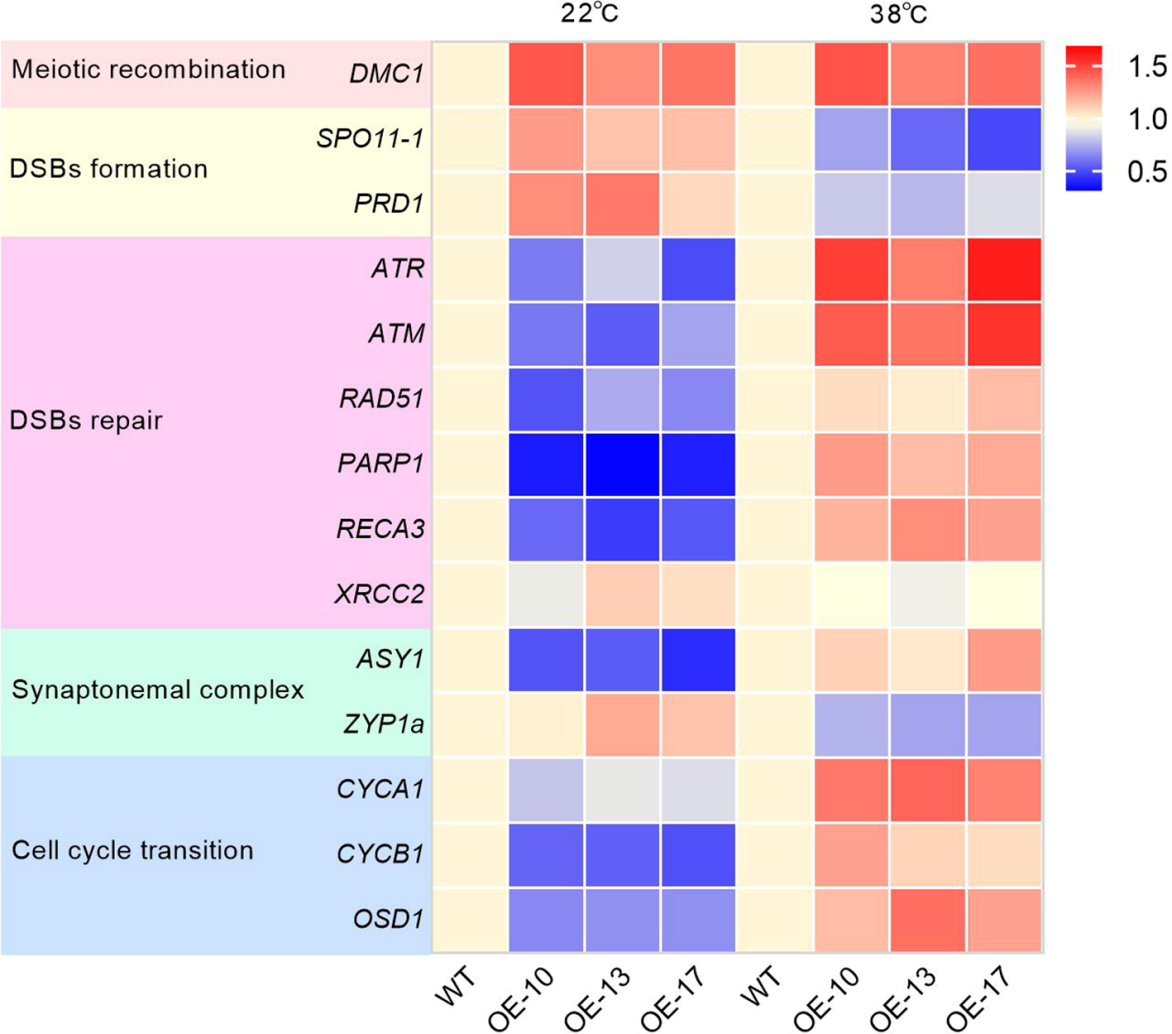
Expression pattern of genes related to DSBs formation, DSBs repair, synaptonemal complex, and cell cycle transitions during meiosis under normal temperature and heat-temperature conditions.

To investigate the molecular basis of *BrDMC1* tolerance to high-temperature stress, we examined the expression of meiosis-related genes. Under normal conditions, the expression levels of *SPO11–1* and *PRD1* required for DSB formation increase, whereas the expression levels of *ATR*, *ATM*, *RAD51*, and *RECA3* required for DSB repair decrease and that of *XRCC2* remains steady. *ASY1* and *ZYP1a*, two parts of the synaptonemal complex, are reduced and unchanged, respectively. Furthermore, there is a decrease in the expression levels of *CYCA1*, *CYCB1*, and *OSD1*, which regulate cell cycle transition (**Figure 7**). High temperature stress reduces the expression levels of *SPO11–1* and *PRD1*, which are required for DSB formation, while increasing the expression levels of *ATR*, *ATM*, *RAD51*, and *RECA3*, which are required for DSB repair, as well as the expression levels of cell cycle transition genes *CYCA1*, *CYCB1*, and *OSD1* (**Figure 7**). These findings suggest that *BrDMC1* responds to high-temperature stress via the DNA repair pathway and cell cycle transition, which improves plant stress tolerance.

## 4 Discussion

With the acceleration of global climate change, regular severe temperature events have emerged as serious environmental threats to crop productivity. Brassica crops, which are key oil and vegetable crops, are highly susceptible to temperature fluctuations throughout growth and development. Meiosis, a critical mechanism in sexual reproduction, is very subject to temperature stress, which directly affects gamete formation and seed development quality (Lei et al., 2020).

### 4.1 DNA repair and antioxidant regulation mechanisms of DMC1

High temperature has a significant impact on plant reproductive development, particularly meiotic diseases, and *BrDMC1* ensures fertility by maintaining the normal meiotic process. In this study, a large number of abnormalities such as lagging chromosomes, chromosome bridges, and unequal segregation were observed in WT pollen mother cells at high temperatures, whereas the incidence of these abnormal phenotypes was reduced in OE-*BrDMC1* lines (OE-10/OE-13/OE-17), and pollen fertility was consistently improved (**Figures 2**, **3**). This is consistent with wheat study results, where *TaDMC1-D1* deletion mutants demonstrated lower meiotic crossovers and higher chromosome synapsis failure rate at 30°C (Draeger et al., 2023). In *A.thaliana*, high temperature suppresses DSB formation, lowers DMC1 activity, and impairs synaptonemal complex formation by altering the chromosomal axis (Ning et al., 2021). These findings suggest that DMC1, by regulating meiotic chromosomal processes, serves as an important barrier for *B.rapa* to survive high-temperature damage. Further cytological observations revealed that the tapetum of OE-*BrDMC1* lines (OE-10/OE-13/OE-17) degraded prematurely under high temperatures (**Figure 4**), but pollen fertility and abnormal chromosomal behaviors were superior to those of WT (**Figure 3**), indicating that *BrDMC1* selectively protects germ cells while prioritizing the protection of key meiotic processes over vegetative tissues. This is consistent with the result that LIM15, the DMC1 homolog in *lilium*, retains meiotic stability under high temperature (Ahn et al., 2025).

ROS eruption in plant cells under high-temperature stress causes membrane lipid peroxidation, protein denaturation, and genomic damage (Bokszczanin and Fragkostefanakis, 2013), and *BrDMC1* also regulates the activity of the antioxidant system, which helps to maintain ROS balance. In addition to its role in homologous recombination and DNA repair, studies have discovered that RecA is also involved in the response to oxygen and heat stress, and the loss of RecA affects survival rate in response to heat shock and desiccation (Aranda et al., 2011). Overexpression of the *ZmRAD51A* in rice boosted the plant disease resistance, resulting to higher expression of defense-related genes (Liu et al., 2019). Under salt stress conditions, *BrDMC1*-RNAi plants exhibit decreased antioxidant enzyme activity, ROS generation, and seed germination rate (Wang et al., 2023). The study observed that OE-*BrDMC1* lines (OE-10/OE-13/OE-17) had considerably lower levels of O_2_^•−^, H_2_O_2_, and OH^•^ following 38°C high-temperature treatment (**Figure 6**), suggesting that BrDMC1 may regulate intracellular ROS homeostasis through direct or indirect pathways to prevent oxidative damage. The activities of SOD, POD, and CAT in OE-*BrDMC1* lines (OE-10/OE-13/OE-17) increased dramatically (**Figure 6**), demonstrating that these antioxidant enzymes work together to constitute the primary intracellular ROS scavenging mechanism. This is comparable to the prior observation in cabbage, which responds to a rise in ROS by boosting antioxidant defense under heat treatment (Soengas et al., 2018).

*BrDMC1* creates a molecular defense network at high temperatures by activating DNA damage repair and cell cycle control genes. The qPCR research revealed that the expression level of *SPO11–1* in OE-*BrDMC1* lines (OE-10/OE-13/OE-17) plants was downregulated, while the expression levels of *ATR*, *ATM*, and *RECA3* genes were dramatically increased(**Figure 7**). SPO11 is responsible for DSB creation, while ATR and ATM are involved in DNA damage repair (Chen and Weir, 2024). Furthermore, high temperatures increase the expression of the *CYCA1* and *OSD1*. CYCA1 not only contributes in the transition from meiosis I to meiosis II, but also, coupled with OSD1, in the prophase transformation of meiosis I (d’Erfurth et al., 2010). The increased expression of these genes suggests that *BrDMC1* may respond to high-temperature stress via these mechanisms, which include improving the formation and repair capacity of DNA double-strand breaks, activating DNA damage detection and signal transduction pathways, regulating cell cycle progression to ensure normal meiosis, and increasing homologous recombination capacity to maintain genome stability. In maize, HSP101 improves homologous recombination repair efficiency by boosting the loading of RAD51 at DSB sites, thus sustaining meiotic stability under high temperature (Li et al., 2022). *Brassica napus* transcriptome analysis under heat stress revealed that upregulated genes are primarily linked to heat shock proteins (HSPs) and other heat shock transcription factors, which play significant roles in heat tolerance (Kourani et al., 2025). Furthermore, HSPs can operate as molecular chaperones to prevent protein denaturation and aggregation, aid refold denatured proteins, and improve plant stress resistance (Zhang et al., 2022). Whether HSPs affect the function of *DMC1* in meiosis under high temperature requires further research.

### 4.2 Functional conservation and evolution of DMC1

*DMC1* is a gene discovered by screening a meiotic cDNA library in *Saccharomyces cerevisiae*. It is a meiosis-specific protein with structural similarities to the bacterial strand exchange recombinase RecA (Bishop et al., 1992). Meiotic recombination is initiated by programmed DSBs, generating single-stranded DNA ends at the break sites, DMC1 performs homology search by polymerizing on single-stranded ends to form nucleoprotein filaments, and conducts strand invasion and strand exchange between homologous chromosomes (Xu et al., 2023). DMC1 requires ATP to participate in homologous recombination, DNA repair, and genomic stability, and it executes DSB repair utilizing homologous chromosomes as templates during meiosis (Crismani et al., 2013; Klimyuk and Jones, 1997).

*DMC1* homologs are found in a range of species. In yeast and mice, *dmc1* mutants exhibit meiotic recombination anomalies, accompanied by chromosome synapsis problems, and cells halt at prophase, resulting in sterility (Bishop et al., 1992; Pittman et al., 1998; Yoshida et al., 1998; Bannister et al., 2007). In medaka *dmc1* mutants, although synaptonemal complex formation is faulty and death occurs in testes, some spermatocytes complete meiosis and generate a modest number of sperm (Chen et al., 2016). In most diploid plant species, disruption of *DMC1* causes sterility. *A.thaliana* possesses a single copy of *DMC1*, and synapsis failure occurs during meiosis I in *Atdmc1* mutants, indicating aberrant crossovers and lower fertility (Couteau et al., 1999). Rice has two *DMC1* homologs, *DMC1a* and *DMC1b*, and single mutants of either show no meiotic defects, however double mutants fail to generate bivalents, eventually resulting to full sterility (Wang et al., 2016). Barley has a single *DMC1* homolog, *HvDMC1*, and mutation of this gene causes synapsis failure, chromosomal bridges, and chromosome fragments at anaphase and telophase of meiosis I and II (Colas et al., 2019; Szurman-Zubrzycka et al., 2019).

In addition to its roles in meiotic homologous recombination and DNA repair, DMC1 plays a role in abiotic stress responses. High temperature in wheat lowers CO formation, which is particularly obvious in *Tadmc1* mutants, demonstrating that the meiotic gene *TaDMC1* is responsible for maintaining proper crossover creation under high temperature (Draeger et al., 2023). Studies have also discovered that *DMC1* maintains seed germination under salt stress by modulating antioxidant enzyme activity and DNA repair gene expression (Wang et al., 2023). In *A.thaliana*, the number of DMC1 foci reduces under heat stress, with no significant change in expression level, although *RAD51* expression declines (Ning et al., 2021). This suggests that high temperatures may limit DMC1 accumulation by altering DMC1 affinity for chromatin via RAD51 (Cloud et al., 2012; Kurzbauer et al., 2012). The decreased number of DMC1 foci could also be a result of impaired DSB generation and/or unstable chromosomal axis (Sanchez-Moran et al., 2007; Ferdous et al., 2012). Unlike *lilium*, LIM15 (a DMC1 homolog) is promptly triggered in anthers at 40°C with dramatically elevated expression levels (Ahn et al., 2025). These discrepancies could be due to species-specific responses to heat stress; for example, dicotyledonous plants like *A.thaliana* may adapt to high temperatures by inhibiting DSBs rather than meiotic repair pathways. Although our study focused on transcriptional expression, subsequent protein-level studies would provide more information on individual responses.

**Figure 8:**
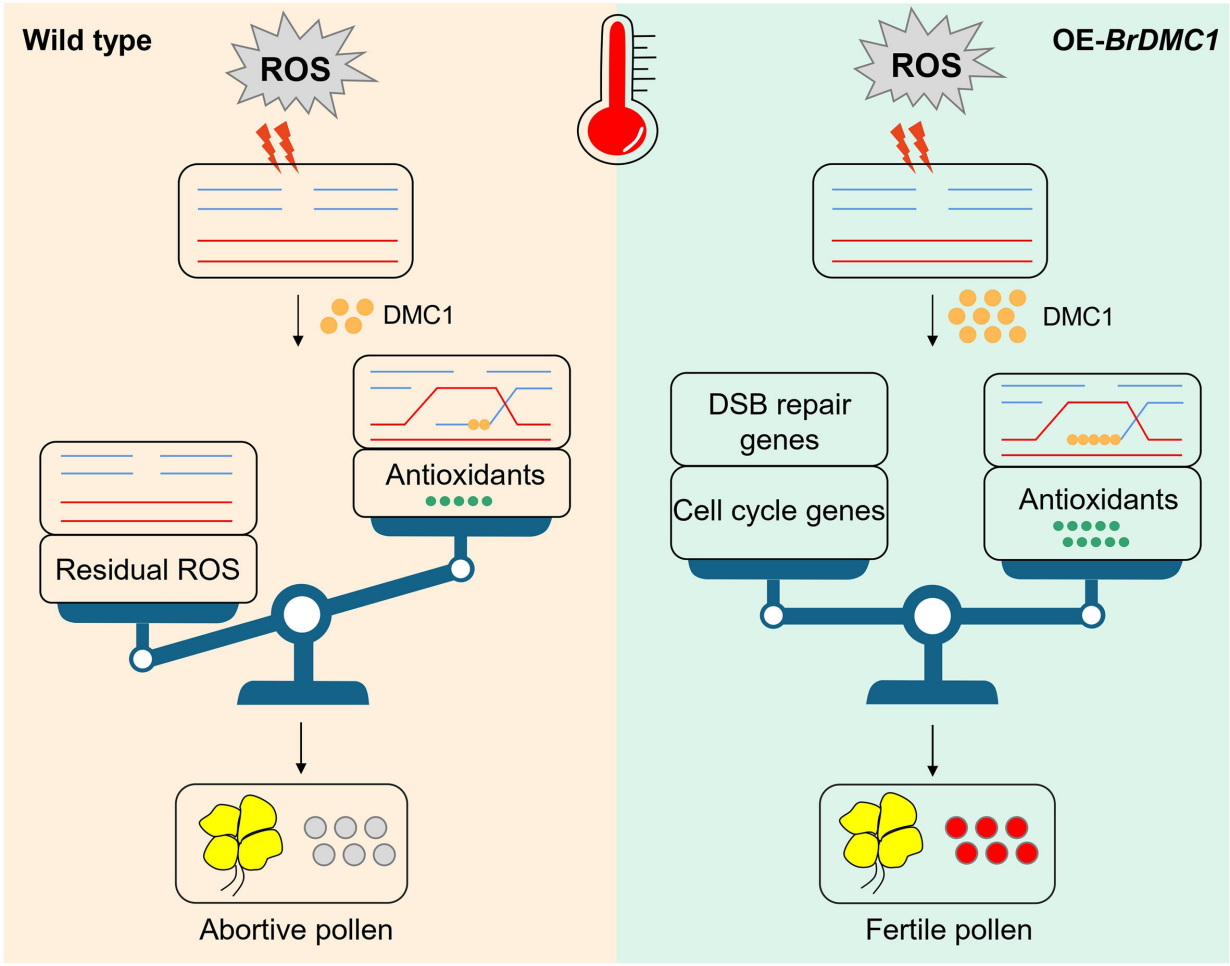
A DMC1 model in *B.rapa* at high temperatures. When plants are subjected to severe temperature stress, they produce a huge amount of ROS, which causes DSBs in the plant. In WT, the fundamental antioxidant repair system is active, but it is unable to properly remove excessive ROS, resulting in ROS accumulation in the plant and inadequate DNA repair, ultimately leading to aberrant pollen formation. In OE-*BrDMC1*, the highly expressed Br*DMC1* not only significantly promotes antioxidant synthesis and efficiently removes ROS, but it also up-regulates the expression of DSB repair genes and cell cycle genes, achieving steady-state regulation of DNA damage repair and the cell cycle and, ultimately, producing fertile pollen.

Based on our findings, we propose a model in which *DMC1*-overexpressed plants respond to high-temperature stress via antioxidant mechanisms and cell cycle transitions (**Figure 8**). In this model, high temperatures cause elevated ROS in plants, resulting in a large number of DSBs. Plants engage fundamental antioxidant mechanisms, however due to poor repair effectiveness, unrepaired DSBs accumulate and residual oxidative damage persists, eventually resulting in abortive pollen. The OE-*BrDMC1* restores homeostasis by activating antioxidant mechanisms and controlling DSB repair genes and cell cycles, resulting in viable pollen.

In conclusion, this study found that DMC1 can positively modulate heat tolerance during meiosis. In natural environments, high temperatures are frequently accompanied by drought stress. Current research focuses primarily on single-temperature stress. In the future, detailed studies on combined stress can be carried out to give a theoretical foundation for crop breeding against abiotic stress.

## Data Availability

The raw data supporting the conclusions of this article will be made available by the authors, without undue reservation.
