# Supplementary material for: BrDMC1-mediated tolerance during pollen meiosis under heat stress in Brassica rapa
Source: Front Plant Sci. 2026 Mar 10;17:1777430. doi: 10.3389/fpls.2026.1777430 (PMC13008927; doi:10.3389/fpls.2026.1777430)
Supplement: Supplementary file 1 [file DataSheet1.docx]

Supplementary Material

# Supplementary Figures

## Supplementary Figure


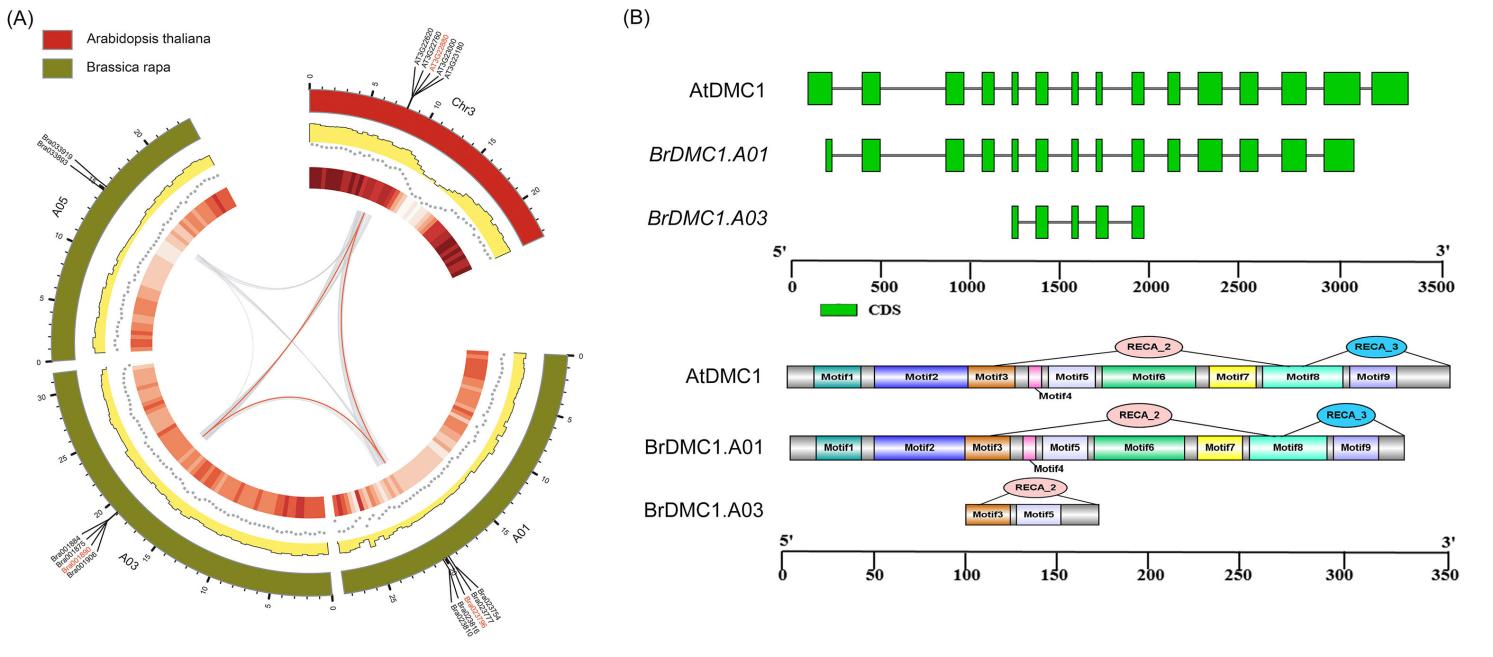


**Supplementary Figure 1.** Bioinformatics analysis of the *DMC1* gene. (A) Collinear correlations of *BrDMC1* genes and neighboring genes in the *A. thaliana* and *B. rapa* genomes. (B) Gene structures, conserved motifs and functional domain of *DMC1*.


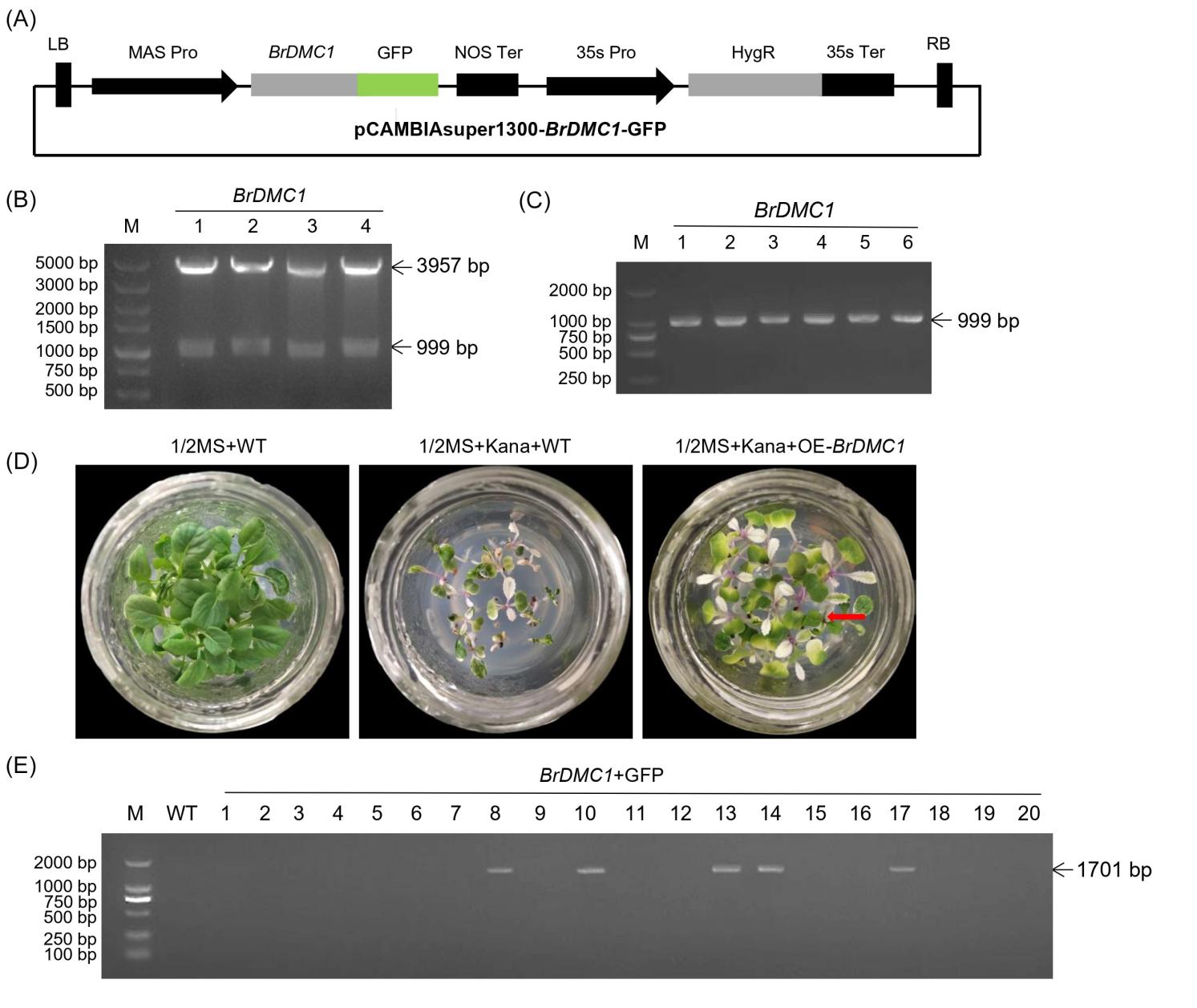


**Supplementary Figure 2.** Overexpression vector construction and transgenic plant examination. (A) An illustration of the pCAMBIAsuper1300-*BrDMC1*-GFP vector's structure.(B) The enzyme digestion map of *BrDMC1* after being ligated to the pEASY-T1 vector. (C) PCR confirmation of the recombinant vector colonies of pCAMBIAsuper1300-*BrDMC1*-GFP. (D) Seed screening using a resistant culture medium. (E) Identification of amplified target fragments in transgenic plants via PCR screening.

# Supplementary Tables

## Supplementary Table

**Supplementary Table 1.** Primers sequence information

| Primers | Sequences (5' to 3') | Gene ID |
| --- | --- | --- |
| *BrDMC1.A01*-F | ATGCTTTCTGCTCTCAAATCGGAA | / |
| *BrDMC1.A01*-R | AAAGGATATAGCTTCGCCTTCAGG |  |
| pro*BrDMC1.A01*-F | ATAAGCAAATGTGTGCTGTGAAC | / |
| pro*BrDMC1.A01*-R | GAAGCGAGCAGAGATCGAAG |  |
| pro*BrDMC1.A03*-F | GTATATGCACACCAAGTGAGACAG | / |
| pro*BrDMC1.A03*-R | CATCAAGAGCTTGGCAACCAG |  |
| *DMC1.A01*-F | GGCCAAAGTTGACAAAATCTG | Bra023796 |
| *DMC1.A01*-R | GCAGTCTCAATCCCACCTC |  |
| *DMC1.A03*-F | GAGAAAATCAGTTGTGCGTATC | Bra001890 |
| *DMC1.A03*-R | AGACTTTAGGAAAGAAACCAGC |  |
| *SPO11-1*-F | ACTGCATTTCCCGTTCCTGT | Bra039374 |
| *SPO11-1*-R | GCCTCTTCCTGTGACAACGA |  |
| *PRD1*-F | TGGAACACCGCAAATCAAGC | Bra000733 |
| *PRD1*-R | ATGGACAGACAAAGCGCAGA |  |
| *ATR*-F | GCTCACACCACAGCAGTTTG | Bra030327 |
| *ATR*-R | GAGAGCTTGCCTGGGTCAAT |  |
| *ATM*-F | GATCTGTATTGCCGGGGCTT | Bra019530 |
| *ATM*-R | GCTCTCAAACAACCGTGCAG |  |
| *RAD51*-F | CAGATTACCTCTGGCTCCCG | Bra006580 |
| *RAD51*-R | TGTTCCCTCGGCATCAATGT |  |
| *ASY1*-F | AAAGACACCACGGAGCGAAT | Bra004222 |
| *ASY1*-R | CGAACTTTCGTCTGGTTGGC |  |
| *ZYP1a*-F | ATCTATGGCGCTTGGATCGG | Bra003654 |
| *ZYP1a*-R | GCGCCAATTCAAGATCGGAC |  |
| *CYCA1*-F | CTTGAGGCGCTTTGTTCGTG | Bra015671 |
| *CYCA1*-R | TTGTGCCAAGAAAACGGCAG |  |
| *PARP1*-F | CTCTGCAACGACGCAAACAA | Bra000883 |
| *PARP1*-R | ATCCACTGATCCAACACCGC |  |
| *RECA3*-F | GGTTGCTAATTCTCGGACGC | Bra001288 |
| *RECA3*-R | ACGCACACATTCAGACCACT |  |
| *XRCC2*-F | ACTTGCATCCTCCCGAAGAC | Bra037778 |
| *XRCC2*-R | GCTCCATTGCCTATCCGGTAA |  |
| *CYCB1*-F | GACGATGACAAGAACGCTGC | Bra011769 |
| *CYCB1*-R | TTTGGCTTTGGTACGGCAAC |  |
| *OSD1*-F | GTACCCAAGGACGCCTCTAC | Bra007362 |
| *OSD1*-R | TTGTGCTCATCTTCCGCCAA |  |
